# The efficacy of a cyclin dependent kinase 9 (CDK9) inhibitor, FIT039, on verruca vulgaris: study protocol for a randomized controlled trial

**DOI:** 10.1186/s13063-019-3570-6

**Published:** 2019-08-09

**Authors:** Takashi Nomura, Eriko Sumi, Gyohei Egawa, Saeko Nakajima, Eiko Toichi, Ryuji Uozumi, Harue Tada, Takayuki Nakagawa, Masatoshi Hagiwara, Kenji Kabashima

**Affiliations:** 1 Department of Dermatology, Graduate School of Medicine, Kyoto University Faculty of Medicine, 54 Shogoin-Kawahara-cho, Sakyo-ku, Kyoto, 606-8507 Japan; 2 Institute for Advancement of Clinical and Translational Science (iACT), Kyoto University Hospital, 54 Shogoin-Kawahara-cho, Sakyo-ku, Kyoto, 606-8507 Japan; 3 Department of Dermatology, Kyoto Medical Center, National Hospital Organization, 1-1 Fukakusa-Mukaihata-cho, Fushimi-ku, Kyoto, 612-8555 Japan; 4 Department of Clinical Pharmacology and Therapeutics, Kyoto University Hospital, 54 Shogoin-Kawahara-cho, Sakyo-ku, Kyoto, 606-8507 Japan; 5 Anatomy and Developmental Biology, Graduate School of Medicine, Kyoto University Faculty of Medicine, 54 Shogoin-Kawahara-cho, Sakyo-ku, Kyoto, 606-8507 Japan

**Keywords:** Cyclin dependent kinase 9 (CDK9), Human papilloma virus (HPV), Verruca vulgaris, FIT039

## Abstract

**Background:**

Human papilloma viruses (HPVs) infect squamous epithelial cells and form verrucous lesions, or warts. Besides the psychosocial problems caused by the disfiguring warts, a subset of HPVs can be the primary etiologic agent for malignancies such as cervical cancer. However, there is no curative antiviral therapy for HPV infection. We recently found that the viral RNA transcription of DNA viruses requires cyclin dependent kinase 9 (CDK9) in the host cells, and that FIT039, a specific inhibitor of CDK9, suppressed the proliferation of DNA viruses such as herpes simplex virus 1 (HSV-1), HSV-2, human adenovirus, human cytomegalovirus, hepatitis virus B, and HPVs. Here, we describe a protocol to evaluate the safety and antiviral effect of FIT039 on common warts in human skin.

**Methods and design:**

A multi-institutional, single-blind, placebo-controlled randomized phase I/II clinical trial was designed to evaluate the safety and efficacy of FIT039 on common warts on the extremities. A total of 44 adults with a primary diagnosis of verruca vulgaris on the extremities without serious comorbidities will be randomized into either the intervention group with an FIT039-releasing transdermal patch or a control group for a duration of 14 days. Outcomes will be assessed at baseline and postintervention. Participants will be further assessed at 2 months follow-up. The primary endpoint for efficacy is the resolution of the warts. The safety endpoint is the incidence of adverse events and adverse drug reactions. The secondary endpoints are changes in the dimensions of the wart, the cross-sectional area of the wart, and the number of clots within the area of the wart.

**Discussion:**

This study is the first to assess the efficacy of FIT039 and will contribute to the development of antiviral agents that can cure refractory common warts in immunocompromised patients.

**Trial registration:**

UMIN Clinical Trials, UMIN000029695. Registered on 1 November 2017.

**Electronic supplementary material:**

The online version of this article (10.1186/s13063-019-3570-6) contains supplementary material, which is available to authorized users.

## Background

Human papilloma viruses (HPVs) are a large group of DNA viruses that infect squamous epithelial cells causing the aberrant proliferation of the infected cells [1]. The most common effect of HPV infection is the formation of warts, or verrucae, on the skin and mucosa of the oral, laryngeal, or anogenital areas. The recurrence of and disfigurement by the warts imposes a considerable psychosocial problem [2]. A subset of HPVs, such as HPV-16 and HPV-18, are designated as high-risk types and are recognized as the primary etiologic agent for cervical carcinoma and its precursor lesions, a subset of the malignancies at other anogenital sites and in the upper aerodigestive tract, and squamous cell carcinoma of the digits [2]. In immunocompromised patients, HPV infections persist and result in an increased risk of developing anogenital neoplasia [2].

The high prevalence of high-risk genital HPV infection is a serious concern because no effective antiviral regimen exists. A human vaccine is now available with more than 90% efficacy for the prevention of type-specific genital HPV infection and the development of associated dysplasia [2–5]. However, vaccination is only effective for a subset of HPV and is ineffective for the established HPV infection. Furthermore, the safety of HPV vaccination is debated in Japan, although the clinical evidence in other countries indicates it is safe [6, 7].

There is no specific antiviral therapy to cure HPV infection. Thus, the focus of the current therapies is to damage or destroy the infected epithelium [8]. Damage to the epidermis can be produced by chemical means, such as salicylic acid, or by physical means, including cryotherapy with liquid nitrogen [8]. A meta-analysis of clinical trials of salicylic acid formulations versus placebo showed that the former significantly increased the chance of the clearance of warts at all sites (risk ratio (RR) 1.56, 95% confidence interval (CI) 1.20–2.03) [9]. A meta-analysis of cryotherapy versus placebo for warts at all sites favored neither intervention nor control (RR 1.45, 95% CI 0.65–3.23) [9]. Topical salicylic acid and cryotherapy are the most common approach to treating skin warts. However, their efficacies are not high, and the patients often experience recurrences. Chemicals such as cantharidin, formaldehyde, glutaraldehyde, phenol, podophyllin, pyruvic acid, and trichloroacetic acid may be effective but are not recommended due to their caustic effects [8]. Evidence is not sufficient for other chemicals such as citric acid, formic acid, silver nitrate, zinc oxide, and zinc sulfate [8].

Another approach to treat warts includes topical application of antiproliferative agents such as bleomycin and 5-fluorouracil (5-FU) [8]. Bleomycin is a cytotoxic agent used in systemic chemotherapy. To treat warts, bleomycin is injected into the affected skin. This procedure is painful, and the pain persists for up to 48 h. Postinflammatory pigmentation can even occur. Topical 5-FU blocks DNA synthesis and damages the basal layer cells of the skin. Topical and intralesional 5-FU produces inflammation and, occasionally, erosions. Hyperpigmentation or hypopigmentation can occur. With the presence of such adverse effects, these agents are not recommended as first-line drugs.

Damaging or destroying the infected epithelium can also induce cell death and antigen exposure and presentation, thereby potentially inducing an immune response [8]. In line with this concept, contact immunotherapy after initial sensitization with diphencyprone or squaric acid dibutyl ester aims to provoke an immune response to warts [8]. The toll-like receptor 7 agonist imiquimod induces a proinflammatory response in the applied area and is effective for genital and perianal warts [8]. Histamine H2 receptor agonists are known to increase the expression of interleukin (IL)-2 and interferon (IFN)-γ in T cells and are sometimes used to treat warts [8]. However, it remains unclear how much the immune response contributes to wart clearance [8]. Furthermore, immunotherapy would not be effective for immunocompromised patients.

Cyclin dependent kinase (CDK) family members are known as important cell cycle regulators [10, 11]. CDK9 is a component of positive transcription elongation factor b (P-TEFb), which phosphorylates the C-terminal domain of RNA polymerase II [11]. As viral RNA synthesis is regulated by P-TEFb, drugs targeting the function of CDK9 could be effective antiviral agents [11]. FIT039 (N-[5-fluoro-2-(1-piperidinyl) phenyl] isonicotinthioamide) selectively binds to the catalytic subunit of CDK9 and specifically inhibits the activity of CDK9 in a dose-dependent manner [10]. Indeed, we have shown that FIT039 suppressed the replication of a broad spectrum of DNA viruses through inhibition of viral mRNA transcription [10]. FIT039 inhibited the replication of herpes simplex virus (HSV)-1 and HSV-2 [10], human adenovirus [10], human cytomegalovirus (CMV) [10], HIV [12], and hepatitis B virus (HBV) [13] in cultured cells. In vivo, the topical application of FIT039 suppressed skin lesions in a murine HSV-1 infection model [10], and intravenously injected FIT039 dramatically enhanced the effect of entecavir in HBV-infected mice [13]. FIT039 neither affected the cell cycle progression of the host cells nor showed toxicity in vivo at the effective dosage. That is because other CDKs can compensate for the function of CDK9. Therefore, We think FIT039 can be used as an antiviral agent for clinical therapeutics. As we recently found that FIT039 suppresses the proliferation of HPV in the raft culture, we are now performing a clinical trial to examine the efficacy of FIT039 on common warts in the skin [14].

### Objectives

The main objective of this study is to examine the antiviral effect of the CDK9 inhibitor FIT039 in the treatment of verruca vulgaris, or warts, caused by HPV. A further objective is to evaluate the safety of an FIT039 transepidermal patch.

## Methods and design

### Study design

**Fig. 1:**
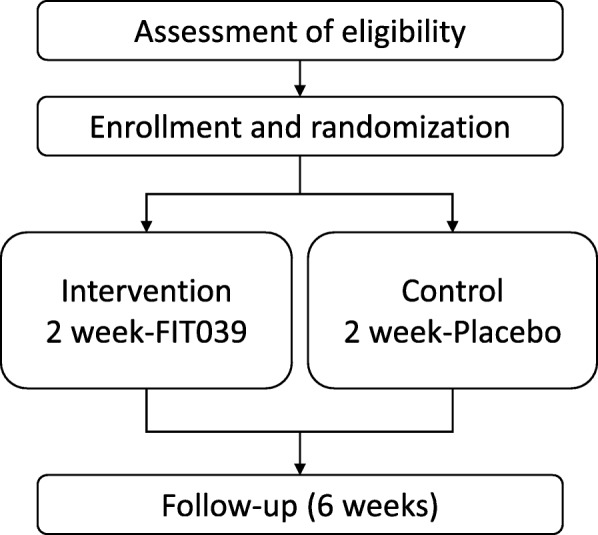
A flowchart of the study design. Eligible subjects will be randomly divided into two groups (intervention and control) and will be followed for 6 weeks

This study is a multi-institutional, single-blind, placebo-controlled randomized phase I/II clinical trial. A total of 44 adults will be enrolled after they provide written informed consent. Participants will be randomly allocated into FIT039 patch or placebo groups in a 1:1 ratio, and will be blinded as to which agent they receive. The target warts of the participants in the FIT039 group will be treated with conventional liquid nitrogen cryotherapy followed by FIT039 patch application for 14 days, with evaluation of the effect of FIT039 for 56 days. The participants in the placebo group will be treated and evaluated the same as those in the FIT039 group except that a placebo patch will be applied. The study is a single-blind trial since the FIT039 patch is distinguishable from the placebo patch by careful comparison for the investigators. The scheme of this study is summarized in Fig. 1. The study was approved by the institutional review board (IRB) at Kyoto University Hospital on 18 October 2017 (K037, protocol version 1.0, September 2017) and the National Hospital Organization Kyoto Medical Center on 22 November 2017. Any protocol modification will be approved by the IRBs before its implementation. This study will be conducted in compliance with the study protocol, the Helsinki declaration [15] and the Ministerial Ordinance on Good Clinical Practice (GCP) for Drugs. This study was registered with the UMIN Clinical Trials Registry as UMIN000029695 (https://upload.umin.ac.jp/cgi-open-bin/ctr_e/ctr_view.cgi?recptno=R000033930).

### Setting

The safety of an FIT039 patch when applied for 14 days on common warts has not been investigated; academic hospitals where at least one investigator-initiated clinical trial in accordance with GCP has been conducted will be recruited. This study is conducted at the Kyoto University Hospital and at the National Hospital Organization Kyoto Medical Center in Kyoto, Japan.

### Eligibility criteria

#### Inclusion criteria

Men and women who meet the following criteria will be included: 1) having verruca vulgaris measuring 4 to 10 mm on the major axis on the extremities, including the palms and soles; 2) aged over 20 years old at the time of consent; 3) a blood test within 4 weeks prior to the registration fulfills all of the following: hemoglobin level ≥ 10 g/dL, white blood cell count ≥ 2000/μL, platelet count ≥ 100,000/μL, aspartate aminotransferase ≤ 100 IU/L, alanine aminotransferase ≤ 100 IU/L, total bilirubin ≤ 2.0 mg/dL, and serum creatinine ≤ 2.0 mg/dL; 4) all men have agreed to use proper contraception during the entire FIT039 patch application period and for 7 days afterwards; 5) women having no possibility of being pregnant and satisfying one of the following conditions, i.e., in menopause, with 12 months or more having passed since the last menstruation without pathological reason, and having undergone permanent contraceptive surgery in the past, such as bilateral oophorectomy and bilateral ligation of the fallopian tubes; and 6) be willing to participate in the study and provide written informed consent.

#### Exclusion criteria

Men and women who meet the following criteria will be excluded: 1) allergic to transdermal patches; 2) having one of the following complications: serious heart disease, gastrointestinal disease, liver disease, kidney disease, systemic skin disorders with lesions at the target verruca vulgaris, uncontrolled diabetes mellitus, infectious diseases requiring continuous treatment by injection or oral application of medical agents, or diseases requiring continuous systemic administration of immunosuppressants or steroids; 3) having an active malignancy, excluding conditions such as adequately treated basal cell carcinoma, intraepithelial carcinoma, or superficial bladder carcinoma, or a malignant tumor not showing metastasis or recurrence for 5 years or more from the end of treatment; 4) having other verruca vulgaris warts within 1 cm from the target wart; 5) have been treated with cryotherapy for the target wart within 4 weeks prior to the acquisition of written consent; 6) taking general antiviral drugs within 4 weeks prior to the acquisition of the written consent; and 7) participating in other trials within 4 weeks prior to the acquisition of the written consent.

### Recruitment

Participants are recruited by advertising on a webpage, advertising inserts distributed with newspapers, and bulletin board advertisements in Kyoto University Hospital. A call center receives contact from respondents to the webpage or advertising inserts and checks their eligibility via a telephone prescreening. If an applicant seems to meet the requirements of eligibility criteria, he/she is invited to contracted clinics to check whether the lesion is a common wart or not.

At the study sites, investigators will obtain informed consent from potential participants and fully examine their eligibility.

Access to personal information about potential participants will be limited to only the investigators and clinical research coordinators.

### Intervention

A verruca vulgaris wart will be treated with liquid nitrogen cryotherapy for 3 s using CRY-AC-3 B800 (Brymill Cryogenic Systems, Ellington, CT, USA) with a nozzle tip of #102-C (aperture 0.022 inches 0.56 mm). All investigators participating in the study are dermatologists who have been guided in advance on how to perform the prescribed cryotherapy in the study protocol. The wart will then be sealed with a 1-cm square transdermal patch that does or does not release FIT039 on day 1. The patch will be covered by transparent waterproof film (OPSITE®; Smith & Nephew, London, UK) to prevent detachment. The patch will be replaced with a new one once a day by the participant until day 14.

The FIT039 patch contains 216 μg FIT039 in a 1-cm square. The FIT039 patch was manufactured at a contract manufacturing organization (CMO) in accordance with Good Manufacturing Practice (GMP) Ordinance for investigational drugs. As FIT039 is a new compound with an antiviral effect, the FIT039 patch has not yet been approved for any indication in any country.

The placebo patch is the same 1-cm square patch as the FIT039 patch but it contains no FIT039. The ingredients of the placebo are equal to that of the FIT039 patch except for FIT039. The placebo patch was prepared using the same manufacturing methods as the FIT039 patch in accordance with GMP.

### Discontinuation

The intervention may be discontinued when the target wart disappears before the first application of the patch, or if the investigator decides to discontinue the intervention due to adverse event(s) or any other reason, or the participant requests discontinuation.

### Adherence assessment

Participants are asked to record the start and end time of each application on a daily basis. Participants are asked to bring the diary and used patches to the hospital to compare the number of used patches with the recorded number of patches in the diary.

### Concomitant medications

Any medicinal, immunological, topical or surgical treatment on the target wart is prohibited throughout the study period except for the cryotherapy directed in the study protocol. However, when a wart has a rough surface like a caldera, cutting the surface of the wart is permitted if it is done before enrollment. Any investigational drug, medical device or regenerative medicine is also prohibited during the study period.

### Measurements

Digital dermatoscopic images of the target wart will be taken by a Derma9500S-GR (Derma Medical Inc., Yokohama, Japan) combined with a G800 digital camera (RICOH, Tokyo, Japan) without and with transparent jelly (Aquasonic 100 gel, Fairfield, NJ, USA) on days 1, 8, 15, 29, 43, and 57. Other evaluations will be carried out according to Table 1.

**Table 1:**
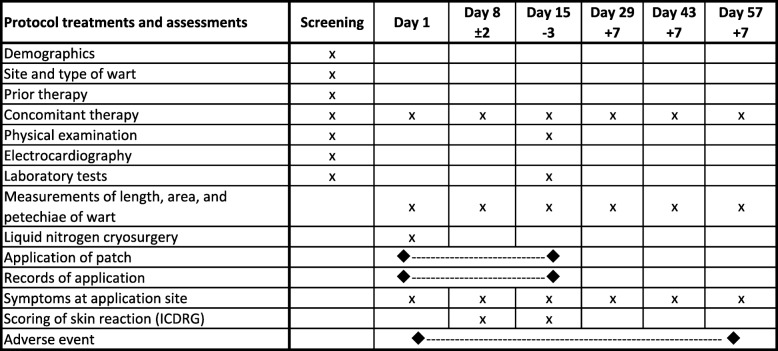
Study schedule

*ICDRG* International Contact Dermatitis Research Group

The primary endpoint of efficacy is resolution of the treated wart, which will be evaluated by the investigators. The safety endpoint is the incidence of adverse events and adverse drug reactions. Adverse skin reactions are graded according to the criteria of the International Contact Dermatitis Research Group (ICRDG).

**Fig. 2:**
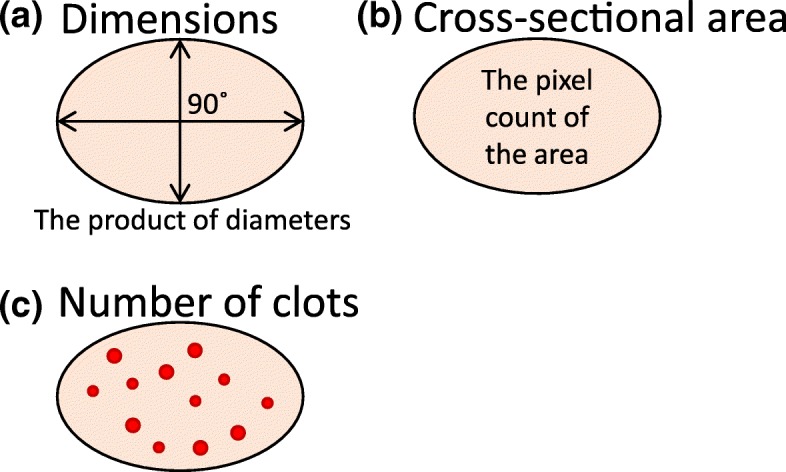
The secondary endpoints are illustrated. **a** Dimensions of a wart are the product of the largest diameter (mm) on an axis line (AL) of the wart and the largest diameter on a normal line of the AL. **b** The cross-sectional area of a wart is calculated from the pixel count of the digital image. **c** The number of clots observable through the surface of a wart is counted

The secondary endpoints are as follows: change in the dimension of the treated wart, change in the cross-sectional area (CSA) of the treated wart, and change in the number of clots within the area of the treated wart lesion. These parameters are calculated based on pictures created by the digital camera dermatoscopic system (Fig. 2).

The dimensions of a wart in this study are defined by the product of the largest diameter (mm) on an axis line (AL) of the wart and the largest diameter on a normal line of the AL (Fig. 2a). Thus:

Change in dimensions = (dimensions of the wart on a subsequent day (mm^2^)) / (dimensions of the wart on day 1 (mm^2^)) × 100 (%).

The CSA is an area (mm^2^) of the target wart that can be calculated by correcting the pixel count of a circumscribed area on a digital image (Fig. 2b). As this is a single-blind study, the area is analyzed using ImageJ by two dermatologists in hospitals other than the institutional sites who are not aware if patients are in the FIT039 or placebo group. Thus:

Change in CSA = (CSA of the wart on a subsequent day (mm^2^)) / (CSA of the wart on day 1 (mm^2^)) × 100 (%).

The change in the number of clots is defined by the change in the number of small blood clots within the area of the wart lesion in some warts, especially of the palmer and plantar type (Fig. 2c). The number of clots will be counted manually by the two dermatologists who are not aware if the patients are in the FIT039 or placebo group. Thus:

Change in clots = (number of clots on a subsequent day) / (number of clots on day 1) × 100 (%).

### Assessment of adverse events

Evaluations of systemic or cutaneous adverse events will be assessed throughout the entire course of the study. Cutaneous adverse events are evaluated in accordance with the scoring system proposed by the ICDRG, as summarized in Table 2 [16].

**Table 2:** Scoring of cutaneous adverse events

Reaction	Scale
No reaction	–
Erythema only (doubtful reaction)	+?
Erythema with infiltration and papules	+
Erythema with edema, papules, and vesicles	++
Formation of bulla(e)	+++
Irritant reaction^a^	IR

^a^Irritant reactions (IR) tend to form a sharply demarcated area, in contrast to a spreading response in allergic reactions, and tend to disappear within several days once the irritating substance is removed

All adverse events will be reported to the data center and handled in accordance with regulatory requirements.

### Data analysis

All efficacy endpoints are assessed for both the full analysis set based on the intention-to-treat principle and the per-protocol set. The full analysis set includes all patients who receive study treatment and who satisfy study inclusion criteria. The per-protocol set includes all patients who receive study treatment for more than 216 h, who adhere to the study protocol, and who do not receive cryotherapy for their target wart prior to day 54. Exact Clopper-Pearson 80% CIs [17] which correspond to a one-sided significance level of 0.10 are calculated with respect to the efficacy primary endpoint of the disappearance of warts for each arm of the study. The remaining efficacy analyses with 95% CIs are considered to be exploratory and are not adjusted for multiple testing. For patients who drop out of this trial regarding the efficacy primary endpoint of disappearance of warts in the efficacy primary analysis, the missing data are imputed as the failure of disappearance of warts (baseline observation carried forward approach). For the other efficacy analyses, missing data are not imputed. The safety endpoint is the incidence of adverse events and adverse drug reactions, assessed for the safety population that included patients who received any study treatment, and summarized by frequency count and percentage. All analyses are performed with the use of SAS statistical software, version 9.4 (SAS Institute, Cary, NC, USA).

### Sample size

The efficacy primary endpoint is the proportion of subjects with resolution of the treated warts as evaluated by the investigators. The Fisher’s exact test is planned at a one-sided significance level of 0.10. A sample size of 44 randomly assigned patients is set to achieve 80% power with consideration for patients who drop out of this trial or who fail to include the full analysis set under the assumption of at least 55% [18–21] disappearance of warts in the FIT039 group compared with a 15% [22] disappearance of warts in the placebo group. A trial with a two-sided significance level of 5% achieving 80% power was not feasible in terms of the required sample size in this phase I/II clinical trial. Therefore, this study was designed to test a hypothesis with a higher significance level, taking into account a reasonable time frame.

### Assignment of intervention

Allocation sequence is prepared by an external Contract Research Organization and is kept strictly confidential by the person in charge of allocation after delivery to the Institute for Advancement of Clinical and Translational Science (iACT), an academic research organization. Therefore, investigators and most staff in iACT remain blinded to the assignment of the intervention for a participant before their enrollment. Stratification factors are the institutions (Kyoto University Hospital or National Hospital Organization Kyoto Medical Center) and the location of the target wart (hand or foot).

Participants will be randomly allocated into the FIT039 patch or placebo patch groups according to the abovementioned allocation sequences at the time when investigators send their patient registration forms to the data center by fax.

Participants are not informed about which group they are assigned. As each FIT039 patch or placebo patch is separately packed with the same label, participants cannot see the difference between the FIT039 patch and the placebo patch. The visits of participants are scheduled on different days so that they do not see other participants. However, the containers of the packed patches have labels indicating whether they contain FIT039 or placebo so that the clinical pharmacist can handle and supply the patches properly.

### Data collection and management

Investigators and clinical research coordinators are advised to fill out paper case report forms following instructions. The completed case report forms will be submitted to the data center at the iACT. The data from case report forms are entered into a database by a double-entry method. Data quality will be validated by checking for missing data and out-of-range values.

Digital dermatoscopic images of the target wart will be also collected and sent to the data center.

In the data center, the data will be stored and handled in a secure server maintaining the anonymity of participants; participants will be identified not by their names, addresses or telephone numbers, but by unique case registration numbers in combination with the date of the investigation.

### Monitoring

An independent data monitoring committee has been established to assess the safety data when serious adverse events may occur and to assess whether the per-protocol set needs any modification.

A qualified and independent auditor is appointed to audit the trial systems and trial conduct before and during the study in accordance with a written procedure.

### Reporting checklist

The SPIRIT reporting guidelines were used to compile the checklist for this protocol [23] (see Additional file 1)

## Discussion

We present the design of a double-arm phase I/II study to evaluate the safety and efficacy of the CDK9 inhibitor FIT039 in the treatment of verruca vulgaris, or common warts. This design will clarify the role of CDK9 in the propagation of the HPV in keratinocytes in humans. The findings of this study will contribute to the further development of antiviral agents.

CDK9 is a component of P-TEFb, which is known to interact with viral factors of various viruses, such as human CMV, Epstein–Barr virus, HIV, human T lymphotropic virus, human adenovirus, influenza A virus, Dengue virus, and Kaposi’s sarcoma-associated virus [11]. Thus, FIT039 may be applicable for a wide spectrum of viruses. Further study is required to evaluate the potential antiviral activity of FIT039.

## Trial status

The trial is ongoing.

## Additional file

Additional file 1 : SPIRIT 2013 checklist: recommended items to address in a clinical trial protocol and related documents. (DOC 126 kb)

## Data Availability

Not applicable.

## Abbreviations

5-FU: 5-Fluorouracil
AL: Axis line
CDK: Cyclin dependent kinase
CI: Confidence interval
CMO: Contract manufacturing organization
CMV: Cytomegalovirus
CSA: Cross-sectional area
GCP: Good Clinical Practice
GMP: Good Manufacturing Practice
HBV: Hepatitis B virus
HPV: Human papilloma virus
HSV: Herpes simplex virus
iACT: Institute for Advancement of Clinical and Translational Science
ICDRG: International Contact Dermatitis Research Group
IFN: Interferon
IL: Interleukin
IR: Irritant reaction
IRB: Institutional review board
P-TEFb: Positive transcription elongation factor b
RR: Risk ratio
